# Network hub centrality and working memory performance in schizophrenia

**DOI:** 10.1038/s41537-022-00288-y

**Published:** 2022-09-23

**Authors:** Hamdi Eryilmaz, Melissa Pax, Alexandra G. O’Neill, Mark Vangel, Ibai Diez, Daphne J. Holt, Joan A. Camprodon, Jorge Sepulcre, Joshua L. Roffman

**Affiliations:** 1 Department of Psychiatry, Massachusetts General Hospital and Harvard Medical School, Charlestown, MA 02129 USA; 2 Department of Radiology, Massachusetts General Hospital and Harvard Medical School, Charlestown, MA 02129 USA; 3 Gordon Center for Medical Imaging, Department of Radiology, Massachusetts General Hospital and Harvard Medical School, Charlestown, MA 02129 USA

**Keywords:** Schizophrenia, Schizophrenia, Human behaviour

## Abstract

Cognitive impairment, and working memory deficits in particular, are debilitating, treatment-resistant aspects of schizophrenia. Dysfunction of brain network hubs, putatively related to altered neurodevelopment, is thought to underlie the cognitive symptoms associated with this illness. Here, we used *weighted degree*, a robust graph theory metric representing the number of weighted connections to a node, to quantify centrality in cortical hubs in 29 patients with schizophrenia and 29 age- and gender-matched healthy controls and identify the critical nodes that underlie working memory performance. In both patients and controls, elevated weighted degree in the default mode network (DMN) was generally associated with poorer performance (accuracy and reaction time). Higher degree in the ventral attention network (VAN) nodes in the right superior temporal cortex was associated with better performance (accuracy) in patients. Degree in several prefrontal and parietal areas was associated with cognitive performance only in patients. In regions that are critical for sustained attention, these correlations were primarily driven by between-network connectivity in patients. Moreover, a cross-validated prediction analysis showed that a linear model using a summary degree score can be used to predict an individual’s working memory accuracy (*r* = 0.35). Our results suggest that schizophrenia is associated with dysfunctional hubs in the cortical systems supporting internal and external cognition and highlight the importance of topological network analysis in the search of biomarkers for cognitive deficits in schizophrenia.

## Introduction

Cognitive impairment is a core feature of schizophrenia and has been linked to poor occupational and social functioning in schizophrenia patients^1–3^. Considering its major impact on functional outcomes and the fact that the available therapeutic tools are limited, cognitive deficits remain an important treatment target. While focal neuromodulation techniques including network-guided transcranial magnetic stimulation (TMS) are increasingly available in psychiatry, the lack of well-defined network targets linking neurophysiology to cognitive outcome renders translational treatment development challenging.

Working memory (WM) impairment is a well-documented cognitive symptom of schizophrenia^4^. WM impairment has been demonstrated in different sensory modalities^5,6^, at various levels of task demands^7,8^ and is typically reflected in reduced WM capacity and slower responses (see prior meta-analysis^9^ for a detailed characterization). While WM deficits in schizophrenia have been thoroughly investigated using imaging and electrophysiology^5,6,10^, there is a paucity of studies investigating WM deficits in the context of brain network topology. Identifying topological features that are associated with cognitive symptoms is important as it could guide targeted treatments.

General functional connectivity abnormalities in schizophrenia have been extensively studied. For detailed reviews, see previous work^11,12^. Whereas structural connectivity and other structural measures are reportedly diminished in schizophrenia^12–15^, functional connectivity has been reported both as increased and decreased, and abnormalities occur in many different regions/networks across the brain^11,12^. This diffuse profile of functional connectivity abnormalities may reflect heterogeneity in study populations and methods, but also the complex nature of schizophrenia pathophysiology. Based on neurodevelopmental models of schizophrenia, genetic and early environmental factors lead to abnormal brain maturation during adolescence and result in a dysfunction in the brain network hubs, which may, in part, explain the diffuse profile of aberrant functional connectivity in schizophrenia^16^. Graph theory provides a robust framework for studying functional brain topology and putatively dysfunctional network hubs in schizophrenia^17,18^.

In the current study, we used a validated, functional network parcellation^19^ and *weighted degree*, a graph theory metric quantifying hub centrality, to identify the cortical hubs that underlie verbal working memory performance in patients with schizophrenia and matched healthy controls. This metric takes both the number of edges connected to a node and the strength of its connections; therefore, it captures the total involvement of the node in the network (i.e., its centrality based on these two features). Unlike many previous graph theory studies focusing on global efficiency in schizophrenia, we sought to determine how disruptions in individual hubs are linked to WM performance. We assessed the correlations between weighted degree and WM performance that are common to both schizophrenia patients and healthy controls as well as those that are unique to schizophrenia. We hypothesized that degree in cortical hubs located in the late-maturing networks in the association cortex such as the default mode network (DMN), frontoparietal and attention networks would be altered in schizophrenia and predict individual differences in WM performance. Finally, in a cross-validated analysis, we built a degree-based predictive model to determine whether WM performance can be reliably predicted using a degree-based summary statistic.

## Methods

### Participants

Forty medicated outpatients with schizophrenia and forty age- and gender- matched healthy controls were recruited as part of a larger fMRI study. Patients were recruited from an outpatient psychosis program at Massachusetts General Hospital. Schizophrenia diagnosis was confirmed with the Structured Clinical Interview for DSM-IV-TR. Results from a separate analysis involving task-related fMRI activation and seed-based functional connectivity on these data have been published elsewhere^5^. Exclusion criteria included history of significant head injury, substance abuse, or neurological disease. Due to the high sensitivity of functional connectivity measures to head motion^20,21^, participants who did not satisfy head motion criteria were removed from the analysis (please see *Preprocessing and Functional Connectivity* for details). Based on these criteria, seven patients were removed. In addition, due to poor image quality, registration between functional and anatomical images failed in three patients, who were also removed. One patient who performed below chance level and showed an inconsistent reaction time pattern was also excluded from the analysis. The remaining 29 patients were matched a priori to 29 participants in the control group based on age and gender and included in the current analysis. Resulting groups were perfectly matched based on gender and closely matched based on age (Table 1). The groups did not differ in head motion (measured by framewise displacement) during the fMRI scan (mean ± standard deviation = 0.108 ± 0.046 for controls and 0.112 ± 0.060 for patients). Information about demographics, use of medication, antipsychotic medication dose and symptom severity of this group of patients is listed in Table 1. All participants gave written informed consent, and the protocol was approved by Partners Human Research Committee. For a correlation analysis assessing the relationships between clinical variables (e.g., positive symptom severity, duration of illness) and working memory performance, see Supplementary Information.

**Table 1:** Demographics, clinical measures, and working memory performance.

	SZ PATIENTS	CONTROLS	*p*
DEMOGRAPHICS	*		
Age	40.6 ± 9.5	40.3 ± 9.5	n.s.
Sex	22 M/7 F	22 M/7 F	n.s.
Race	15 Caucasian/14 Other	19 Caucasian/10 Other	n.s.
Length of illness (years)	16.2 ± 9.4	-	
Handedness	4 Left/25 Right	3 Left/26 Right	n.s.
CLINICAL			
PANSS total	72.9 ± 14.1	-	
Antipsychotic dose (mg CPZE)	632.7 ± 608.8		
Atypical antipsychotics (%)	86.2	-	
Antidepressants (%)	44.8	-	
Anticonvulsants (%)	31	-	
PERFORMANCE			
Estimated Verbal IQ**	100.2 ± 11.8	109.3 ± 10.2	0.003
WM accuracy (% correct)	84.5 ± 10.1	91.5 ± 6.0	0.002
WM reaction time (ms)	949.6 ± 147.7	879.5 ± 129.7	0.059

*CPZE* chlorpromazine equivalent, *PANSS* positive and negative syndrome scale, *WM* working memory, *SZ* schizophrenia, *n.s.* not significant.

*Mean ± SD is shown for quantitative variables.

**Verbal IQ was missing for two patients.

### Working memory task

The Sternberg Item Recognition Paradigm^22^ (SIRP) was used to evaluate WM function at 4 different loads (1, 3, 5 and 7 letters). The SIRP is a verbal WM task that has been shown to induce robust linear changes in reaction time and brain activation in response to increasing load^5,22,23^. EPRIME 1.1 was used to present stimuli and collect the participants’ responses. All participants performed a practice run to become acclimated to the task. Each block of the WM task involved encoding, delay and multiple probe epochs (Fig. S1). During encoding, participants were asked to memorize a set of 1, 3, 5 or 7 consonants displayed on the screen. Then the delay epoch was introduced with a fixation cross (2 s), which was followed by the presentation of 14 successive probes consisting of letters for 1.1 s, each separated by a varying intertrial interval (ITI). Participants indicated, using a keypad placed in the dominant hand, whether the letter displayed on the screen was a target (one of the letters presented during encoding; 50% of probes) or a foil (not presented during encoding; 50% of probes). Probes were jittered (range 1.7 to 3.6 s) to facilitate a separate event-related analysis. Each of the four task loads was used twice during the task run, totaling 112 trials and 8 blocks.

### MRI acquisition

MRI data were acquired in a 3 T Siemens TIM Trio System using a 12-channel quadrature head coil. A T1 image (repetition time/echo time/flip angle = 2200 ms/1.54 ms/7^o^) with 144 axial slices and a voxel size of 1.2 × 1.2 × 1.2 mm^3^ was acquired. Resting state fMRI images were collected using the following scan parameters: repetition time/echo time/flip angle = 3 s/30 ms/ 85^o^, in-plane resolution = 3 mm × 3 mm, slice thickness = 3 mm. Before the sequence, participants were asked to keep their eyes open and stay still during 6 min of scanning.

### Preprocessing and functional connectivity

Preprocessing was performed using FMRIB Sofware Library (FSL, v6.0, https://fsl.fmrib.ox.ac.uk/fsl/fslwiki), and Matlab v9.10 (https://www.mathworks.com/products/matlab.html). The anatomical image (T1-weighted) was first reoriented to the anterior commissure–posterior commissure (AC-PC) plane and the brain skull was stripped. The image was then segmented into gray matter, white matter, and cerebrospinal fluid and normalized to the Montreal Neurological Institute brain template (MNI152). The first four volumes in the fMRI series were removed to ensure the stabilization of the MR signal. The preprocessing of resting state fMRI data included realignment using the middle functional volume and head motion correction using a six-parameter rigid body linear transformation, and intensity normalization. Given the sensitivity of functional connectivity measures to head motion, a quality control analysis was performed using Art Repair (https://www.nitrc.org/projects/art_repair/) and customized scripts^24^. Framewise displacement (FD) was computed at each timepoint. The participants who had average FD > 0.25 mm (over the resting state run) were excluded from the analysis. In addition, volumes with FD > 0.5 mm were scrubbed from the connectivity analysis (0.7% and 1.7% of all volumes in controls and patients respectively). All functional images were normalized to the MNI152 brain template (3 mm^3^ isotropic), smoothed with a 6 mm full-width-at-half maximum (FWHM) isotropic Gaussian kernel and band-pass filtered retaining BOLD signal between 0.01 Hz and 0.08 Hz. Potential confounders were removed via regression by applying a model including the 6 parameters from rigid body linear transformation, the global signal, and applying the component-based method CompCor (5 parameters from cerebrospinal fluid signal and 3 parameters from the white matter signal). The residual volumes were used for the functional connectivity analysis.

Regions of interest (ROIs) were defined using the Gordon et al. parcellation, which includes 333 parcels generated using surface-based resting state functional connectivity boundary mapping^19^. These parcels are assigned to 12 distinct networks: default mode (DMN), visual (VN), frontoparietal (FP), dorsal attention (DAN), ventral attention (VAN), salience (SN), cingulo opercular (CON), somatomotor (SM), somatomotor lateral (SML), auditory (AN), cingulo parietal (CP), and retrosplenial (RSP). Based on this parcellation, communities with very small number of parcels (<5) are not assigned to a separate network and named ‘unassigned’ (UA). Functional connectivity was computed as the correlation between each parcel’s mean signal time course and every other parcel’s mean time course using Pearson’s product moment correlation. 333 × 333 correlation matrices were converted to z-maps using Fisher’s z transformation, to enhance the normality of the distribution of correlations. We then removed all negative correlations to minimize ambiguity in interpretation^25,26^. All ROI analyses were performed in the volume space and projected onto the surface for visualization using Connectome Workbench^27^.

### Degree centrality

**Fig. 1:**
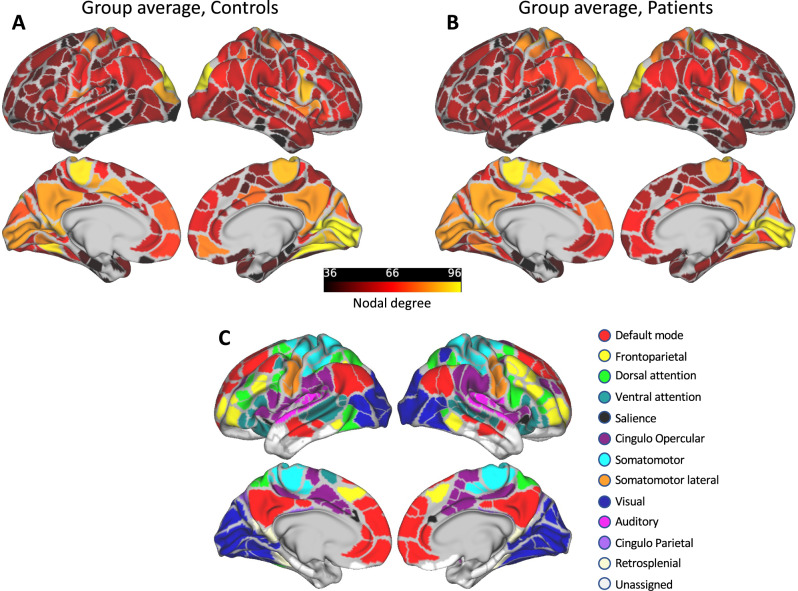
Weighted degree per group. Group averaged composite weighted degree is illustrated for controls (**A**) and schizophrenia patients (**B**). Gordon et al. parcels used in this analysis and their corresponding color-coded networks are displayed in (**C**). Areas with high degree centrality included nodes in the primary sensory, motor, and default mode networks in both groups.

The processing pipeline for weighted degree centrality computation is illustrated in Fig. S2. Following published methods^28^, we used a range of sparsity thresholds between 2–10% edge density (with 1% increments) to construct the graphs and obtain a robust estimate of degree centrality. Degree values from each thresholded matrix were summed up to obtain an estimate of ‘composite’ weighted degree per ROI for each subject. This step ensures that the resulting networks are robust and do not rely on a particular threshold. Weighted degree at each threshold was computed as the sum of all weighted connections to a node in the thresholded matrix^29^:

$$k_i = \mathop {\sum }\limits_{j = 1}^n a_{ij}$$

where *a*_*ij*_ is the correlation between the time series of i^th^ and j^th^ parcel, n is the total number of parcels, and k_i_ is the weighted degree for the i^th^ parcel. Figure 1A, B depicts the group average composite weighted degree for each of the 333 cortical nodes and Fig. 1C displays the network assignment for each of the Gordon parcels. In order to test for significant differences in weighted degree between the groups, we used permutation testing (*n* = 5000) to obtain a null distribution. In this analysis, the group labels were randomly reassigned and the difference between group means were calculated for each permutation. The *p*-value was calculated as the proportion of permuted mean differences that were greater than or equal to the observed difference between the group means. The obtained *p*-values were corrected for multiple comparisons across 333 parcels at 5% False Discovery Rate (FDR).

### Relationship to behavior

We assessed the relationships between weighted degree and two behavioral outcomes, namely WM accuracy (averaged across all WM loads) and reaction time (averaged across all trials and loads) using a correlation analysis. A nonparametric permutation test was utilized to determine the significance of the correlations^30,31^. 5000 permutations were performed to estimate the standard error of the correlation coefficient at each node. The correlations were then converted into *z*-scores (by dividing them by the standard error), which we used to calculate two-tailed *p*-values. FDR at 5% was used to correct for multiple comparisons across 333 parcels. In addition, a threshold of *p* < 0.025 was used for familywise error, which represents a Bonferroni-corrected value for the two behavioral variables (WM accuracy and reaction time). Thus, the correlations with FDR-corrected *p*-values surviving this threshold were considered significantly correlated with the behavioral measure.

### Within- vs. between-network edges

Among the ROIs that showed a significant correlation with behavior exclusively in patients, we further analyzed the connectivity patterns in these nodes. We focused on these ROIs because the presence of correlation uniquely in patients suggests a mechanism involved in the pathophysiology of the impairment. Specifically, within-network degree and between-network degree were calculated separately considering only the edges from the same network as the node and those from different networks respectively. As in our main correlation analysis, degree was obtained for graphs that were defined using a range of thresholds (2–10% edge density). For each of the ROIs, we then calculated the Pearson’s correlation between the behavioral measure (WM accuracy or reaction time) and within- or between-network degree. In this post-hoc analysis, correlations with *p* < 0.05 were considered significant.

### Prediction analysis

To examine the predictive value of degree features on behavior in new individuals, we employed degree-based predictive modeling. Connectome-based predictive models are robust tools that allow to identify brain-behavior relationships with improved generalizability (e.g., compared to simple regression or correlation)^32,33^. Establishing the predictive power of neuroimaging measures can improve translational applicability. In this analysis, we tested whether primary degree features could be used to predict WM accuracy or reaction time of the participants. For this prediction analysis, we utilized our entire sample (*N* = 58) to maximize statistical power for cross-validation. Following previously published methods^32^ and established protocols^33^, we employed leave-one-out cross-validation (LOOCV) in our entire sample (*N* = 58) to (i) select degree features that are relevant for WM performance, (ii) build a summary statistic from these features using the training set, (iii) test the model on the subject that is left out in each iteration, and (iv) and evaluate the power (correlation between predicted and observed values) and significance (permutation testing) of the prediction. For the details of this prediction analysis, please see Supplementary Methods.

## Results

### Behavioral results

**Fig. 2:**
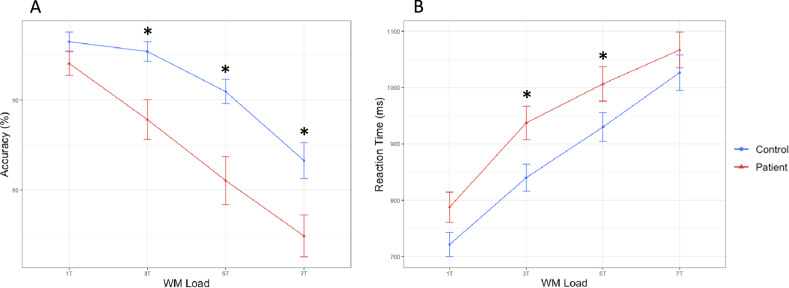
Behavioral results. Percent WM retrieval accuracy (**A**) and average reaction time (**B**) are displayed per WM load for controls (blue) and patients with schizophrenia (red). Patients consistently performed less accurately across working memory loads and responded more slowly compared to healthy controls. Asterisks demonstrate significant differences between the groups (*p* < 0.05).

Figure 2 depicts the behavioral results for both groups at each WM load level. Consistent with previously reported results in a larger version of this cohort^5^, a two-sample *t*-test revealed that patients performed less accurately than controls at WM loads 3, 5, and 7 (*p* = 0.001 for combined accuracy). This analysis also revealed a significant effect in reaction time for moderate WM loads (3 and 5) with patients responding more slowly (*p* = 0.025 for combined reaction time).

### Group differences in weighted degree

Weighted degree was calculated for each of the Gordon parcels using weighted connections and by summing up degree values from a range of graph thresholds. Figure 1 shows the degree maps averaged for each group. Areas with high degree included the primary sensory and motor areas as well as the DMN regions along the medial wall. While none of the ROIs survived the FDR correction, a number of regions showed medium to large effect sizes for group differences in weighted degree. Among the regions with higher weighted degree in controls, the largest effect sizes in group differences were observed in two nodes in right anterior insula (*d* = 0.998, *d* = 0.67), and right postcentral gyrus (*d* = 0.68). Conversely, among the regions with higher degree in patients (vs. controls), the largest effect sizes were found in left intraparietal sulcus (IPS, *d* = −0.98), left lateral orbitofrontal cortex (lOFC, *d* = −0.65), and left superior parietal lobule (SPL, *d* = −0.64). All ROIs showing moderate to large effect sizes in group differences are shown in Fig. S3 for illustration purposes, and the ROIs showing nominally significant group differences and their corresponding Gordon networks are listed in Table S1.

### Relationship between weighted degree and behavior

Association between weighted degree and the two behavioral outcomes (WM accuracy and reaction time) was assessed using a correlation analysis and FDR for multiple comparisons correction. Across all participants, degree in a node in the parahippocampal gyrus (unassigned network) as well as in superior temporal sulcus (STS) showed a significant correlation with accuracy, whereas degree in supplementary motor area (SMA) and fusiform gyrus (FG) showed a significant correlation with reaction time. For illustration purposes, correlation maps for both behavioral outcomes across all participants as well as in each group are displayed in Fig. S4 and Fig. S5 respectively. Table 2 lists the ROIs showing significant correlations with both behavioral outcomes in each group, as well as across both groups.

**Table 2:** Regions showing significant correlations between weighted degree and behavioral outcomes.

Region	Network	Coordinates			Correlation coefficient	p_corr_
		x	y	z		
WM ACCURACY						
All subjects						
STS	VAN	58	−45	9	0.330	0.0183
PHG	UA	32	−9	−36	−0.421	0.0216
Controls						
IPL	DMN	49	−53	29	0.506	0.0015
mPFC	DMN	−7	55	18	−0.474	0.0100
dlPFC	FPN	−43	19	34	−0.484	0.0101
STG	AN	−60	−39	17	0.387	0.0153
Patients						
Precuneus	CON	−17	−36	43	−0.661	4.69E-09
PHG	UA	32	−9	−36	−0.582	5.32E-05
STS	VAN	46	−37	3	0.547	2.77E-04
STS	VAN	47	−22	−9	0.459	0.0025
STS	VAN	61	−39	2	0.444	0.0119
REACTION TIME						
All subjects						
SMA	SM	5	−17	52	−0.442	0.0049
FG	VN	−34	−44	−22	−0.400	0.0083
Controls						
Patients						
dlPFC	DMN	−42	16	48	0.615	1.15E-08
FEF	DAN	−45	3	32	0.577	1.21E-04
IPL	DMN	−47	−58	31	0.508	1.50E-04
SMA	SM	5	−17	52	−0.525	6.11E-04
FG	VN	−34	−44	−22	−0.519	0.0074
IPS	DAN	−43	−45	43	0.553	0.0189
STS	VAN	58	−45	9	−0.536	0.0189
SMA	FPN	−6	29	44	0.469	0.0227
SMG	CON	58	−40	35	0.473	0.0227

*dlPFC* dorsolateral prefrontal cortex, *FEF* frontal eye field, *FG* fusiform gyrus, *IPL* inferior parietal lobule, *IPS* intraparietal sulcus, *mPFC* medial prefrontal cortex, *PCC* posterior cingulate cortex, *PHG* parahippocampal gyrus, *PoCG* postcentral gyrus, *SMA* supplementary motor area, *SMG* supramarginal gyrus, *STG* superior temporal gyrus, *STS* superior temporal sulcus.

Networks: *AN* auditory network, *CON* cingulo-opercular network, *DAN* dorsal attention network, *DMN* default mode network, *FPN* frontoparietal network, *SM* somatomotor network, *UA* unassigned, *VAN* ventral attention network, *VN* visual network.

**Fig. 3:**
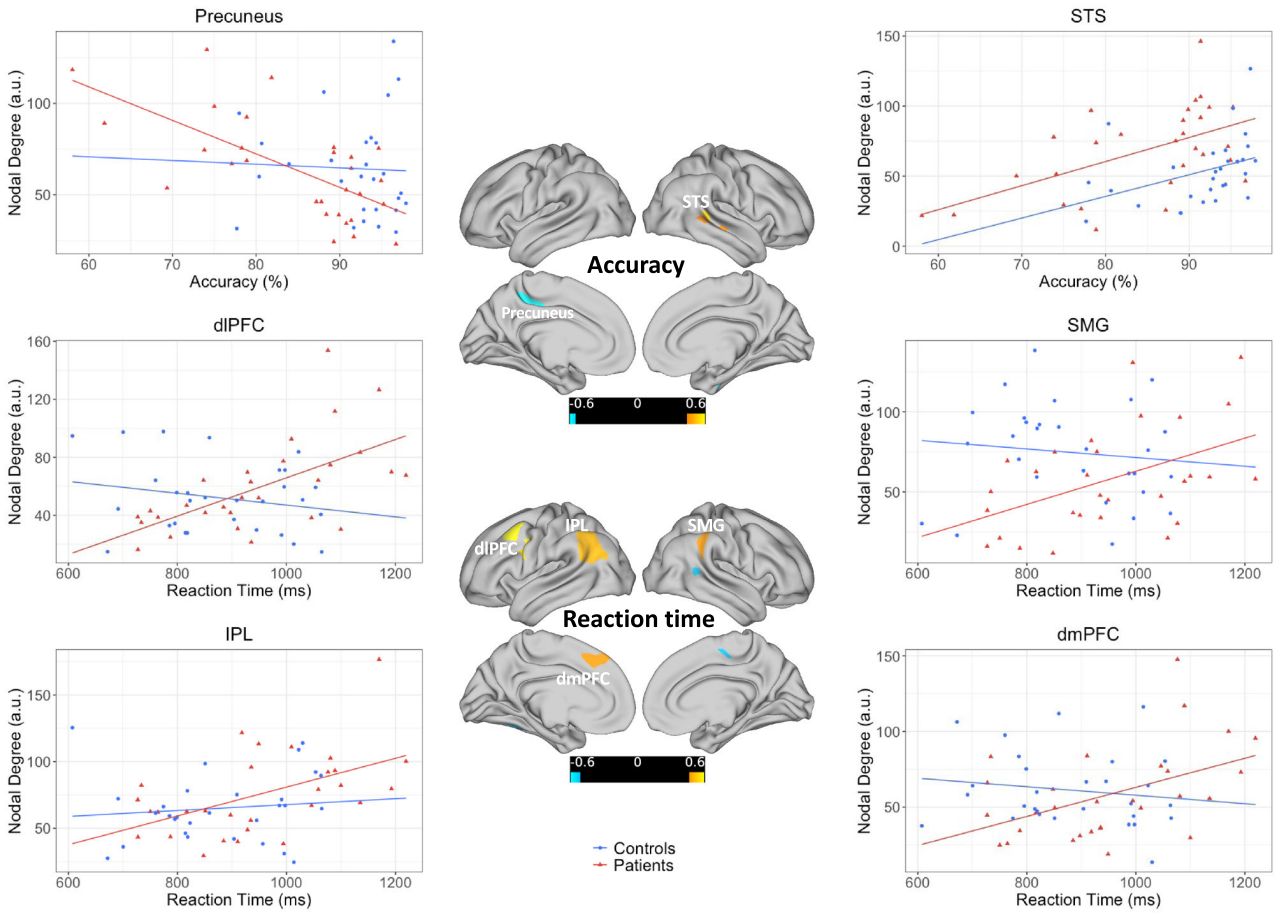
Weighted degree and behavior. Scatterplots depict the relationships between WM performance and weighted degree in six of the fourteen nodes that showed a significant correlation with performance in patients. See Fig. S6 for the remaining nodes. The data points and the best fit lines are plotted in blue for controls and in red for patients. The left and right panels show the relationships with WM performance for the nodes marked on the surface views in the middle panel. Warm colors depicting the regions represent a positive correlation and cool colors reflect a negative correlation with the behavioral measure. The scatterplot is shown for only one of the three neighboring significant nodes in the STS.

In controls, weighted degree correlated negatively with WM accuracy in DMN regions such as mPFC, and in FPN regions such as the dlPFC. Conversely, degree in left superior temporal gyrus and right inferior parietal lobule (IPL) showed a significant positive correlation with accuracy. Figures 3 and S6 depict the scatterplots for all the ROIs that show significant correlations exclusively in patients for each behavioral outcome. In patients, weighted degree in left precuneus and right parahippocampal gyrus (PHG) showed a strong negative correlation with WM accuracy, whereas degree in multiple STS nodes in the VAN was positively correlated with WM accuracy. Higher degree in DMN and DAN regions including left IPL, left dorsolateral prefrontal cortex (dlPFC), and left frontal eye field (FEF) was associated with longer reaction times in patients. Finally, higher degree in posterior SMA, STS, and FG was associated with faster responses in patients.

### Within- vs. between-network

To further investigate the 14 nodes (listed in Table S2 and illustrated on the surface maps in Figs. 3 and S6) where degree was significantly correlated with behavior exclusively in patients, we calculated weighted degree for within-network and between-network edges separately. For 8 of the 14 nodes, the correlation with the behavioral measure remained nominally significant for both within- and between-network degree (Table S2). Two nodes in the DAN (left FEF and left IPS) showed a significant correlation with reaction time only for between-network degree (FEF within-network: *p* = 0.107, FEF between-network: *p* = 0.001; IPS within-network: *p* = 0.213, IPS between network: *p* = 0.0003). Similarly, a node in the VAN (right STS) and another node in the CON (right SMG) significantly correlated with reaction time only for between-network degree (STS within-network: *p* = 0.118, STS between-network: *p* = 0.0007; SMG within-network: *p* = 0.101, SMG between-network: *p* = 0.007). A node in the right PHG showed a significant correlation with WM accuracy only for between-network degree (within-network: *p* = 0.183, between-network: *p* = 0.00003). Finally, a DMN node (left IPL) showed a significant correlation between reaction time and within-network degree (*p* = 0.003), but a correlation at trend-level strength with between-network degree (*p* = 0.076).

### Predicting WM performance

**Fig. 4:**
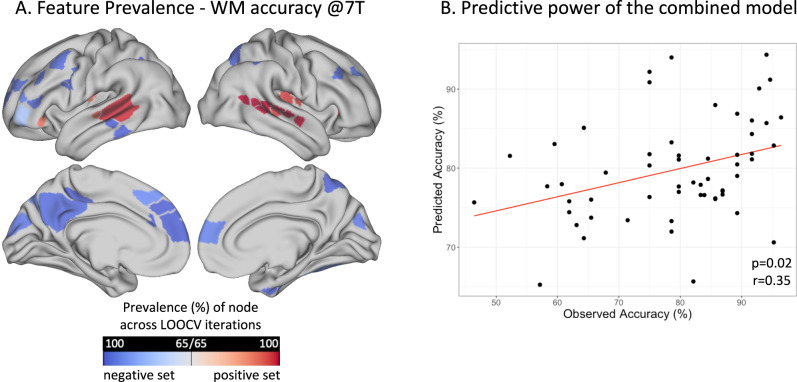
Predictive modeling. Feature prevalence across cross-validation iterations is shown for a significant predictive model that predicted WM accuracy at high load (**A**). This model was built using weighted degree from both groups combined to maximize statistical power (*N* = 58). Nodes with high prevalence scores were consistently selected as primary features correlating with behavior at each LOOCV and contributed to the summary degree score. On the right panel, predicted vs. observed WM accuracy is displayed for this significant model (**B**). Abbreviations: 7T 7-letter task condition. LOOCV Leave-one-out cross-validation.

In a cross-validated analysis, we tested whether a single summary statistic derived from weighted degree can be used to predict individual WM accuracy and reaction time (averaged across all loads). The significance of the predictions was determined via permutation testing. WM accuracy prediction was not significant (*p* > 0.05) for the model using the combined degree score (derived from the positive and negative sets). However, a closer examination of performance at different WM loads revealed that the model was predictive of WM accuracy for the high WM loads (*p* = 0.022, *r* = 0.34 for 5T and *p* = 0.022, *r* = 0.35 for 7T accuracy), but not for the lower loads (*p* > 0.05). The nodes that were predominantly selected across the different iterations of cross-validation for the significant model predicting WM accuracy at 7-letter condition are displayed in Fig. 4. DMN nodes including PCC, mPFC, and dlPFC (from the negative set) as well as the VAN nodes in the STS (from the positive set) were consistently selected across iterations. Reaction time prediction was not significant at individual WM loads or when averaged across loads (*p* > 0.05).

## Discussion

Schizophrenia is increasingly recognized as a cognitive disorder driven by abnormal neurodevelopment and consequent dysfunction in the brain’s network hubs. Therefore, identifying network biomarkers that underpin cognitive performance in patients is crucial for developing novel, targeted treatment strategies. In this study, we utilized a resting state graph theory analysis to assess impairments in network hub function in schizophrenia and determine the relationship between a robust hub centrality index and working memory performance. Our findings revealed diffuse abnormalities, with moderate effect size, in patients with schizophrenia in cortical hubs encompassing top-down control networks such as the FPN and CON, the DMN, somatomotor and attention networks. Our correlation analysis revealed that elevated degree centrality in nodes in the DMN such as left IPL and dlPFC as well as areas of the CON such as precuneus was strongly associated with poorer WM performance in patients. Conversely, higher degree centrality in the VAN regions along STS, fusiform gyrus and posterior SMA was associated with better WM performance. Further, we found that summary degree scores derived predominantly from DMN regions were predictive of WM accuracy in both patients and control subjects. However, within the DMN, we found that largely different cortical locations account for WM performance in patients and controls, which suggests that discrete mechanisms underlie working memory function in patients with schizophrenia and healthy controls. These effects appeared to be unrelated to clinical outcomes such as positive symptom severity, disease chronicity and antipsychotic medication dose, as these variables were not associated with WM performance in patients. This is consistent with previous studies reporting nonsignificant associations between positive symptoms and neurocognitive performance in schizophrenia^34,35^ and emphasizes the distinct role of cognitive impairment in this disorder^36^.

Our findings demonstrated both increases and decreases in weighted degree in schizophrenia throughout the cortex. While these changes have not reached significance at 5% FDR, at uncorrected levels, the direction of the effects was generally consistent in each network. For example, we found that anterior insula (as part of VAN and CON) and areas of the lateral somatomotor network showed reduced degree in schizophrenia patients. Conversely, IPS, angular gyrus, and lOFC encompassing the default mode and frontoparietal networks showed increased weighted degree in patients. Increases in connectivity may be compensatory or reflect dysregulated circuitry due to abnormal hub functioning^16^. Our correlation analysis revealed that regions showing elevated degree in schizophrenia patients (e.g., IPS) predicted poorer performance. This suggests that these effects are less likely to be compensatory and more likely to reflect dysregulated circuitry.

In terms of the relationship between weighted degree and working memory performance, although we observed several common themes among patients and controls (e.g., higher degree in DMN linked to poorer performance, higher degree in VAN linked to better performance), different nodes accounted for behavioral performance in the two groups. For example, the mPFC showed an association with WM accuracy in controls, yet this effect was not present in patients. In patients, two other DMN nodes in the left IPL and left dlPFC exhibited a strong correlation with a different aspect of behavior, reaction time. This dichotomous pattern suggests that functional organization of the DMN and its role in cognitive function is altered in patients. Previous work consistently demonstrated altered DMN connectivity in patients with schizophrenia^37,38^, their first-degree relatives^38^, and individuals at clinical high risk^39^ for schizophrenia. Our findings suggest that DMN is centrally implicated in working memory function in both health and schizophrenia^38,40^, but this relationship manifests itself through different areas of the network.

Anterior insula and ventral attention/salience networks are thought to play a significant role in working memory function putatively by supporting dorsal and ventral visual attention systems^41^ and coordinating other task-related networks to facilitate access to attentional resources^42^. Lower degree in right anterior insula we found in our study might underlie some of the clinical features associated with schizophrenia such as aberrant salience^43^. We did not observe a significant relationship between reduced degree in insula and working memory performance in patients. However, both in controls and in patients, degree in other nodes of the VAN (e.g., superior temporal cortex), exhibited significant correlation with WM accuracy. Moreover, degree scores from these nodes were consistently selected during cross-validation in a significant model predicting WM accuracy at high loads. High frequency oscillations in the superior temporal cortex were previously linked to working memory maintenance in electrophysiology studies in humans^44,45^. Additionally, in a fMRI study using a large cohort of healthy individuals, we previously demonstrated that both within- and between-network connectivity to areas of the VAN are among the primary features predicting specific WM load in a support vector machine classification algorithm^46^. Taken together, the observation that weighted degree in superior temporal areas is associated more strongly with WM accuracy (vs. reaction time) may indicate a specific role in WM maintenance.

Aside from VAN and DMN, nodes in other networks also accounted for WM performance. For example, weighted degree in precuneus, a medial parietal area of the CON, negatively correlated with WM accuracy. While its exact role in WM is not clear, repetitive TMS to the precuneus during the probe phase of a verbal WM task was shown to improve accuracy^47^. A dysregulated hub in this region may contribute to the WM deficits in schizophrenia. We also identified several regions that were exclusively associated with reaction time. Frontal and parietal areas of the DAN such as left FEF and left IPS, as well as right supramarginal gyrus (SMG) showed a positive correlation with reaction time. The dorsal attention system plays an important role in visual processing speed and putatively contributes to age-related decline in processing speed^48^. Therefore, dysregulated nodes in this network in schizophrenia might contribute to slower responses during cognitive performance in patients. Notably, the correlation with reaction time was driven by between-network connectivity for these nodes. IPS and SMG have been shown to underlie the interaction between memory load and visual attention during a spatial WM task^49^. A load-dependent role for these regions may require interaction with other task-related networks including the FPN and DMN, and thus their relationship to behavioral performance may depend on this cross-network talk. Finally, degree in posterior SMA was negatively correlated with reaction time. SMA is implicated in monitoring of both successful and erroneous actions^50,51^, thus, connectivity deficits in this area may impair efficient action monitoring during the probe epochs in our task and slow down responses to the probes.

### Limitations

Our study has several limitations. Due to our predetermined head motion criteria, we had to remove a number of patient participants from the analysis, which reduced our sample size and potentially our statistical power. Similarly, we combined data from controls and patients in our degree-based predictive model analysis to maximize statistical power, which limits the ability to make group-specific inferences. Replication or extension of our findings in other patient samples will provide further clarity on the effects that we demonstrated in our study. In addition, it is not clear if the elevated degree we observed in several networks (e.g., DMN, FPN, limbic regions) in patients reflects abnormal structural connectivity, a compensatory response to structural deficits, or simply the local pathophysiology. Future studies using both functional and structural imaging modalities can be helpful in elucidating this point.

## Conclusion

Patients with schizophrenia showed diffuse abnormalities in hub centrality particularly in VAN, FPN, DMN, SM and cortical limbic areas. Some of these hubs appear to be relevant to WM impairment and account for individual differences in performance. Predictive models using summary degree scores successfully predicted WM accuracy at high load in both groups. DMN and VAN features were predominantly involved in such models. The nodes that were found to be critical to patients’ WM performance in the current study can be examined in future work on cognitive impairment in schizophrenia, and weighted degree can be tested as a potential physiological marker in studies using noninvasive neuromodulation.

## Supplementary information

Supplement

## Data Availability

The data that support the findings of this study are available from the corresponding author upon request.

## Code availability

All Matlab and R scripts that were used in the analyses reported in this manuscript are available from the corresponding author upon request.
