# Supplementary material for: Network hub centrality and working memory performance in schizophrenia
Source: Schizophrenia (Heidelb). 2022 Sep 23;8(1):76. doi: 10.1038/s41537-022-00288-y (PMC9508261; doi:10.1038/s41537-022-00288-y)
Supplement: Supplementary file 1 — Supplement [file 41537_2022_288_MOESM1_ESM.pdf]

Supplementary Information

Network hub centrality and working memory performance in schizophrenia

Hamdi Eryilmaz PhD[1], Melissa Pax, BA[1], Alexandra G. O'Neill, BS[1], Mark Vangel, PhD[2], Ibai Diez, PhD[3], Daphne J. Holt, MD, PhD[1], Joan Camprodon, MD, MPH, PhD[1], Jorge Sepulcre, MD, PhD[3], Joshua L. Roffman, MD, MMSc[1]

[1]Department of Psychiatry, Massachusetts General Hospital and Harvard Medical School, Charlestown, MA 02129
[2]Department of Radiology, Massachusetts General Hospital and Harvard Medical School, Charlestown, MA 02129
[3]Gordon Center for Medical Imaging, Department of Radiology, Massachusetts General Hospital and Harvard Medical School, Charlestown, MA 02129

Contents

Supplementary Methods
Supplementary Results
Supplementary Figures 1-6
Supplementary Tables 1-2

SUPPLEMENTARY METHODS

Clinical and cognitive outcomes

In order to assess the relationship between key clinical variables and cognitive outcomes, we tested whether clinical measures such as positive symptom severity, antipsychotic medication dose and duration of illness were correlated with working memory performance. Positive symptom severity was assessed via Positive and Negative Syndrome Scale (PANSS)[1]. Antipsychotic medication dose information was available for 23 of the 29 patients included in the current analysis. Chlorpromazine equivalent (CPZE) dose was calculated based on previously validated conversion formulas[2]. Duration of illness was considered from the onset of psychotic symptoms to the time of the experiment. Pearson's correlation coefficients were calculated to assess the correlations between these variables and working memory performance (both average retrieval accuracy and reaction time). The resulting p values were Bonferroni corrected for the six comparisons (3 clinical variables and 2 cognitive outcomes) in this analysis.

Degree-based predictive modeling

In this analysis, we used leave-one-out cross-validation (LOOCV) in our entire sample (N=58) to i) select primary degree features associated with behavior in the training set (N=57), ii) build a summary statistic using these features (i.e., degree score), iii) test the model in the subject that is left out, iv) and evaluate the significance of the prediction using permutation testing. At each iteration of the cross-validation, we removed one subject's data (test set) from the rest of the dataset (training set). We then selected the most relevant features in the training set using a significance threshold ($p<0.1$) for the correlation between nodal degree and behavior (WM accuracy or reaction time). The selected features were separated into positive and negative sets depending on the sign of the correlation with the behavioral outcome. Next, a summary degree statistic was computed (for each subject in the training set) by summing up the degree values of the nodes in the feature sets. Thus, a separate summary value was obtained for the positive and negative set. We then produced a combined summary score obtained by subtracting the negative set summary score from the positive set score, which utilizes and takes advantage of both feature sets[3]. This metric represented a summary "degree score". The relationship between degree score and the behavioral variable was modeled using linear least squares regression:

$$BV' = a*DS + b$$

Where *BV* stands for behavioral variable (WM accuracy or reaction time) and *DS* degree score. The models were built for positive and negative sets, as well as the combined set. Finally, the degree score was calculated in the test subject for all three sets, and the behavioral variable was estimated using their respective model. We repeated these steps until each of the 58 participants was used as a test subject. Then, the correlation between the predicted and the observed values of the behavioral variable determined the predictive power of these models. The significance of this correlation was tested via permutation testing. To obtain an empirical null distribution of the correlation, we applied the cross-validated procedure described above using the same degree data and randomly shuffled behavioral scores and repeated this step 1,000 times. Finally, the p-value was calculated as the proportion of permuted correlations that are greater than or equal to the true prediction correlation.

SUPPLEMENTARY RESULTS

Relationship between clinical and cognitive outcomes

Our analysis of correlation between the main clinical and cognitive outcomes in our study revealed no significant relationship between these sets of variables. The correlations between working memory accuracy and the three clinical variables were not significant: r=-0.17, p=0.37 (PANSS positive), r=0.15, p=0.48 (CPZE), and r=-0.18, p=0.36 (illness duration). Similarly, the correlation between average reaction time and the clinical variables did not reach significance (r=0.24, p=0.21 for PANSS positive, r=-0.08, p=0.72 for CPZE, and r=0.08, p=0.70 for illness duration.

SUPPLEMENTARY FIGURES

Figure S1

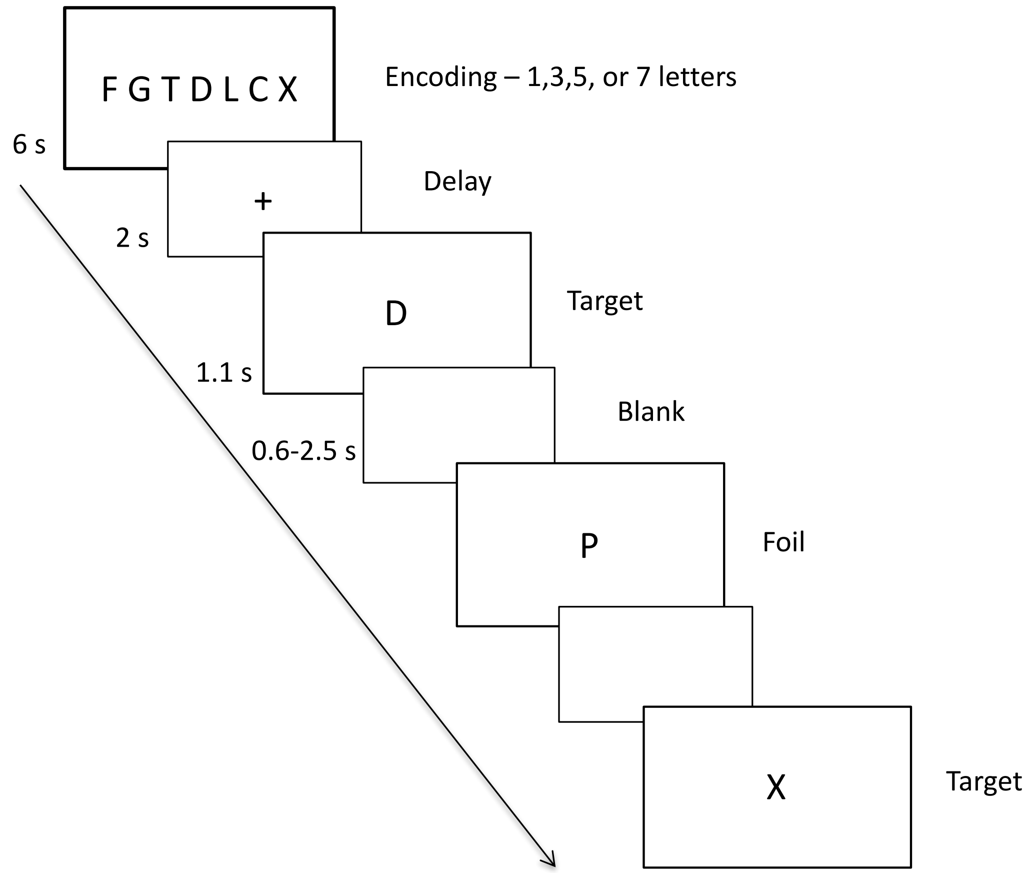

Task. Sternberg Item Recognition Paradigm (SIRP) was used as the working memory task. Each block started with the encoding epoch where participants memorized 1, 3, 5, or 7 consonants. After a brief delay period, single probe letters were presented in succession (14 probes per block). The participants were instructed to press '1' on the keypad if the letter was not in the memorized set (foil) and press '2' if the letter was in the memory set (target). Participants completed 8 blocks (2 per WM load) consisting of 112 trials during a single fMRI run.

Figure S2

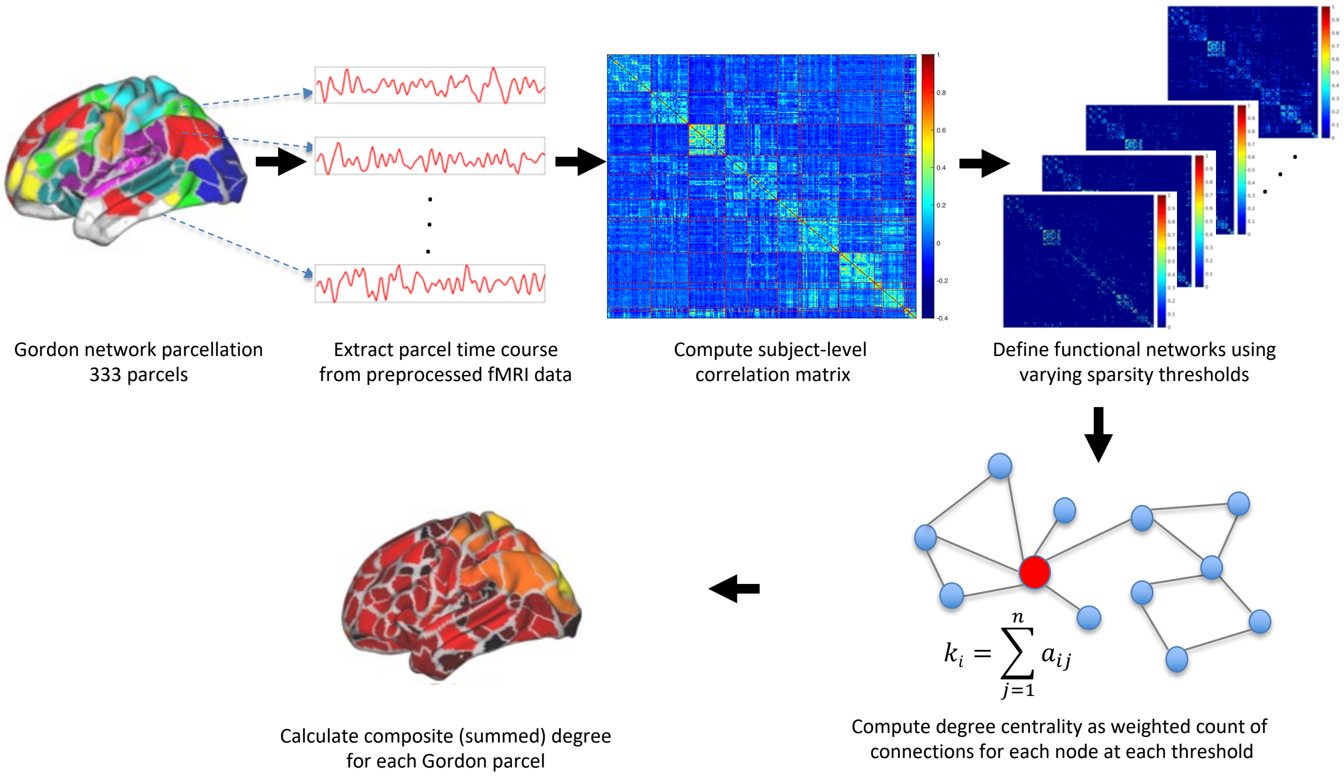

Analysis pipeline. Resting state functional connectivity was computed among 333 Gordon parcels covering the cerebral cortex for each participant. Subject-level connectivity graphs were defined at varying thresholds (from 2% to 10% edge density in 1% increments) to obtain a robust composite degree value at each node.

Figure S3

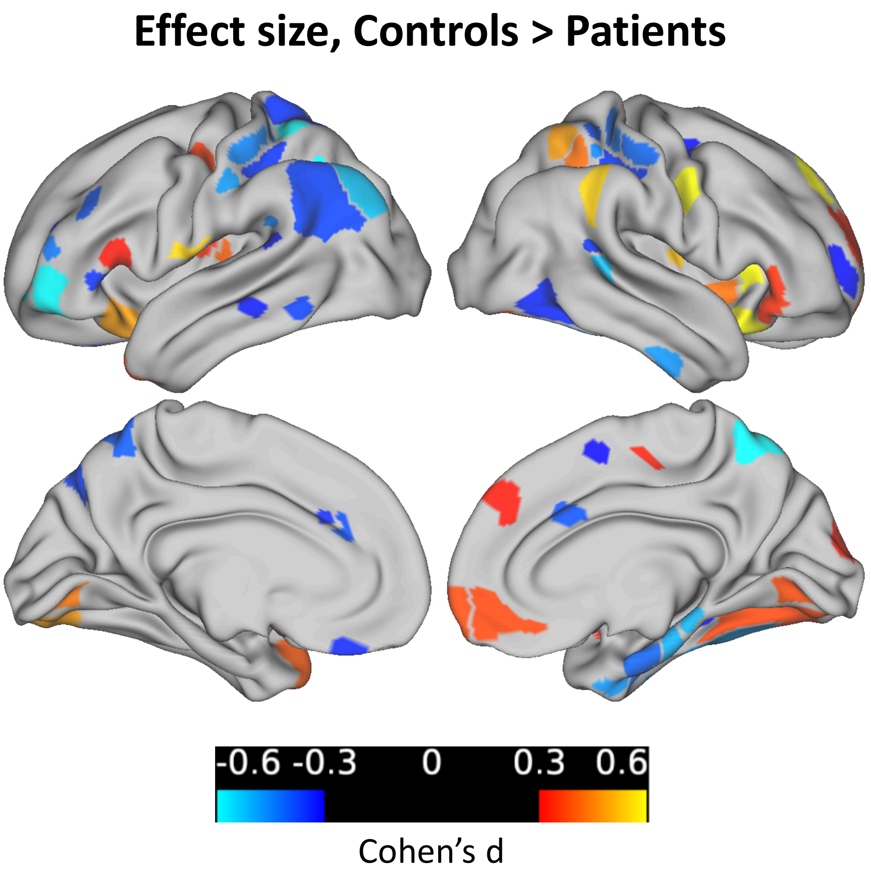

Group difference. Effect size (Cohen's d) of group differences in nodal degree is shown on the surface maps. Warm colors indicate nodes with greater degree value in controls, cool colors indicate nodes with greater degree value in patients. Right anterior insula and right postcentral gyrus showed the strongest effects for lower nodal degree in schizophrenia, whereas left intraparietal sulcus showed the largest effect for higher nodal degree in patients.

Figure S4

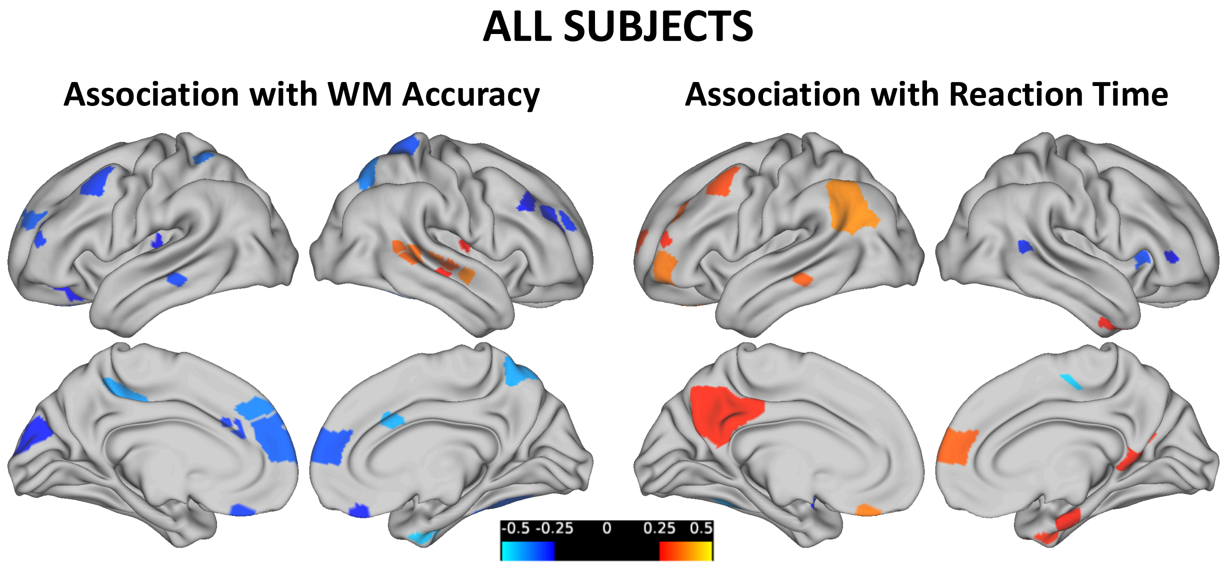

Relationship between nodal degree and behavior across all subjects. Pearson's correlation between weighted degree and each of the WM performance measures is shown on the surface maps. Parahippocampal gyrus and medial default mode network regions showed an inverse relationship between weighted degree and WM performance (i.e., higher degree was associated with poorer performance). Conversely, regions of the ventral attention network showed a positive correlation between nodal degree and WM accuracy.

Figure S5

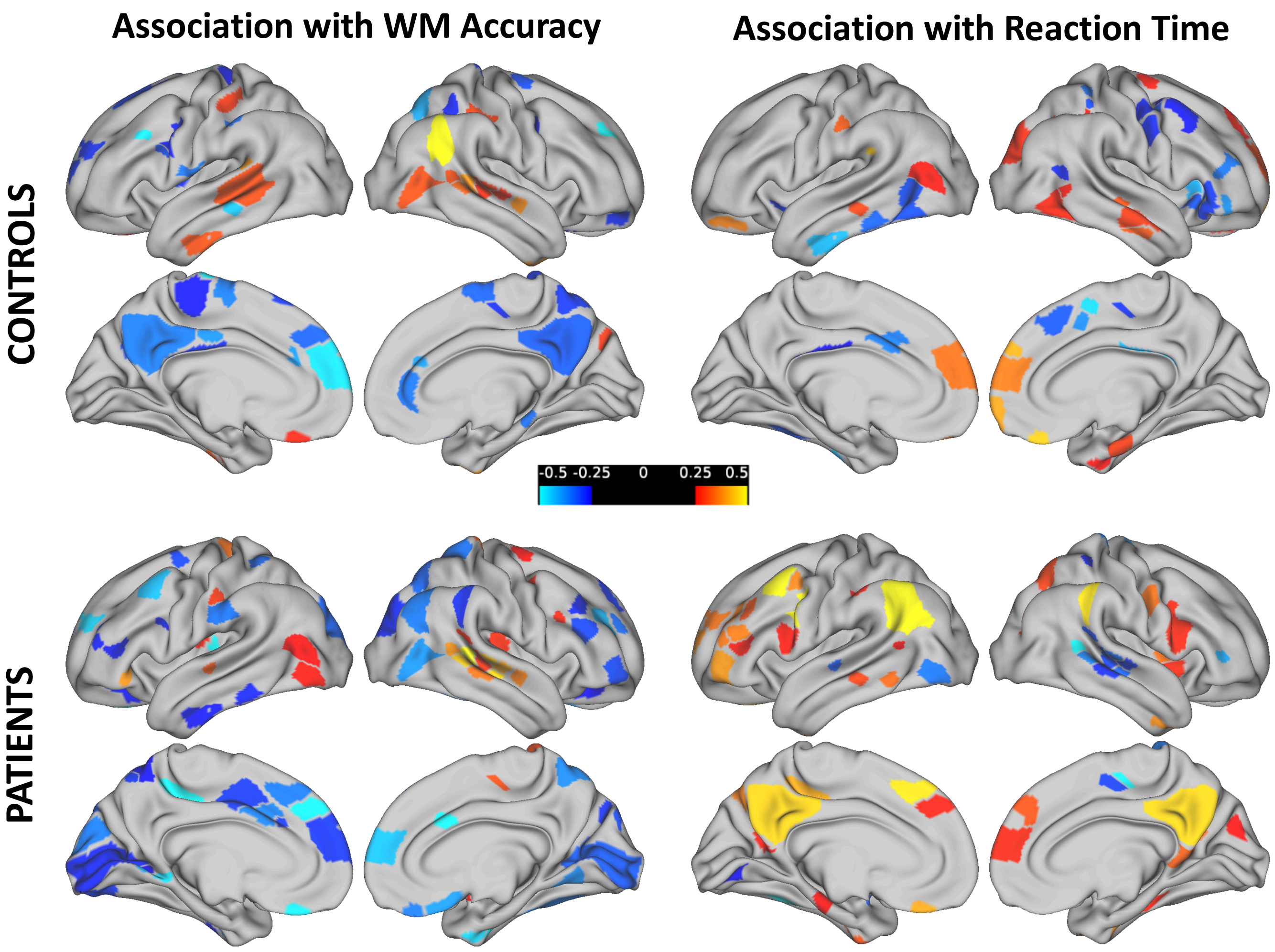

Relationship between nodal degree and behavior in each group. Pearson's correlation between nodal degree and WM performance measures is shown on the surface maps for controls (top) and schizophrenia patients (bottom). Both groups showed positive correlations between accuracy and nodal degree in superior temporal regions. The degree in default mode regions seemed to correlate more strongly with accuracy in controls, whereas it showed a stronger association with reaction time in patients.

Figure S6

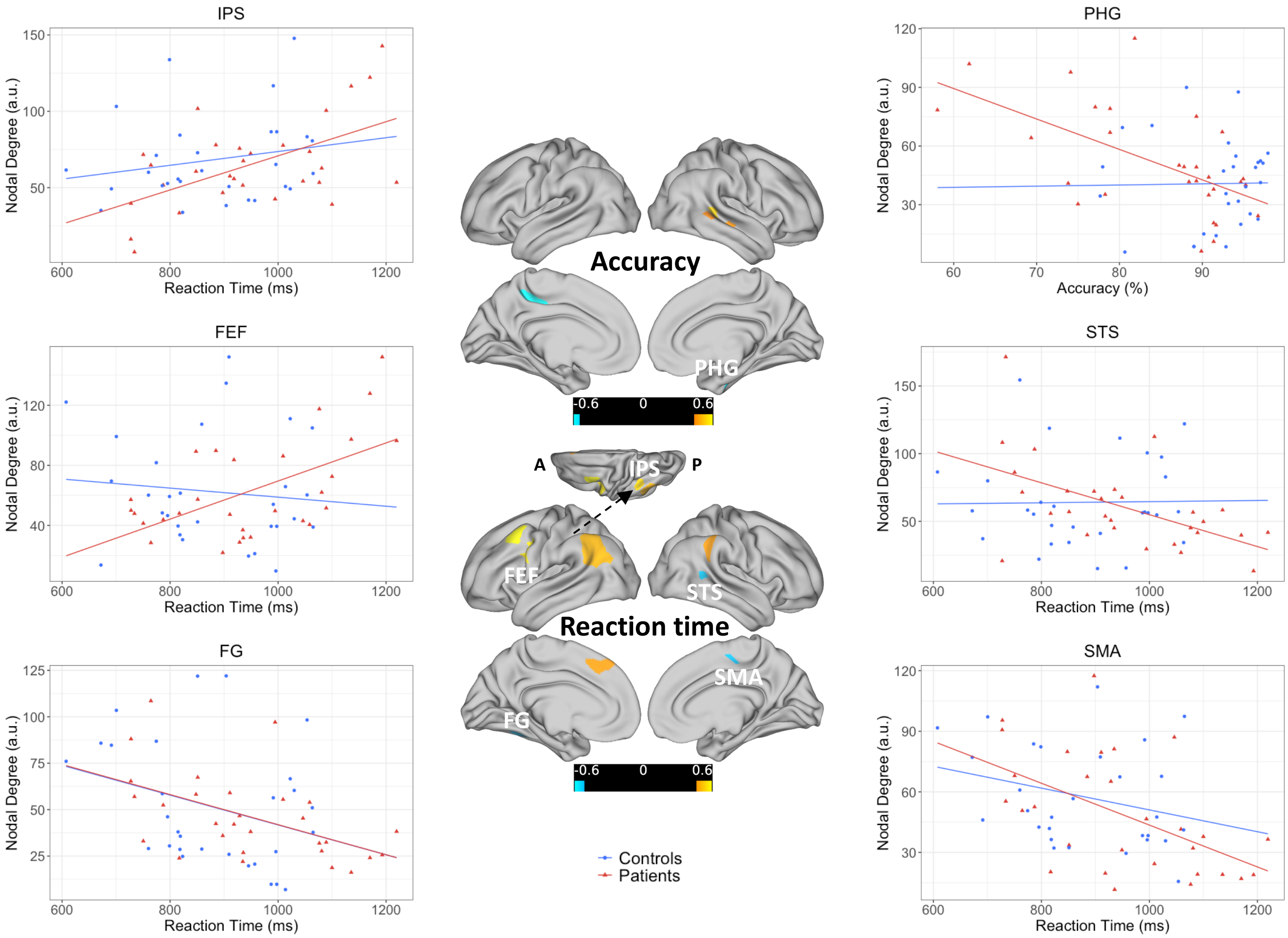

Relationship between nodal degree and behavior in schizophrenia. Scatterplots illustrate the relationships between WM performance and degree in six of the fourteen nodes that showed a significant correlation with performance in patients. See Figure 3 in the main article for the other nodes. The data points and the best fit lines are plotted in blue for controls and in red for patients. The nodes are marked on the surface maps in the middle panel. Warm colors represent a positive correlation and cool colors reflect a negative correlation with the behavioral measure. Abbreviations: A: anterior, FEF: frontal eye field, FG: fusiform gyrus, IPS: intraparietal sulcus, P: posterior, PHG: parahippocampal gyrus, SMA: supplementary motor area, STS: superior temporal sulcus.

SUPPLEMENTARY TABLES

Table S1. Gordon parcels showing nominally significant group differences in nodal degree between schizophrenia patients and healthy controls.

Region	X	Y	Z	Network	p-value (uncorr.)	Cohen's d
IPS	-34	-61	42	FPN	0.0004	-0.977
aIns	27	20	-15	VAN	0.0006	0.998
PoCG	50	-7	36	SML	0.0110	0.679
lOFC	-23	61	-7	UA	0.0148	-0.653
lOFC	-40	50	-5	FPN	0.0148	-0.657
aIns	34	23	4	CON	0.0152	0.670
SPL	-29	-45	62	SM	0.0168	-0.644
dmPFC	14	47	42	DMN	0.0184	0.638
Precuneus	10	-57	58	DAN	0.0186	-0.637
ROp	-60	-4	9	CON	0.0438	0.541
AG	-39	-74	38	DMN	0.0480	-0.528
STS	46	-37	3	VAN	0.0488	-0.523

Negative Cohen's d values indicate higher degree in patients.

Abbreviations: AG: angular gyrus, aIns: anterior insula, dmPFC: dorsomedial prefrontal cortex, IPS: intraparietal sulcus, lOFC: lateral orbitofrontal cortex, PoCG: postcentral gyrus, ROp: Rolandic Operculum, SPL: superior parietal lobule.

Networks: CON: cingulo-opercular network, DAN: dorsal attention network, DMN: default mode network, FPN: frontoparietal network, SM: somatomotor network, SML: somatomotor lateral, UA: unassigned, VAN: ventral attention network, VN: visual network

Table S2. Correlations between behavioral outcomes and within- and between-network nodal degree in schizophrenia patients.

Region	Network	Coordinates			WN correlation	WN p_{corr}	BN correlation	BN p_{corr}
		x	y	z				
**WM ACCURACY**								
Precuneus	CON	-17	-36	43	-0.483	0.008	-0.573	0.001
PHG	UA	32	-9	-36	-0.254	**0.183**	-0.697	2.63E-05
STS	VAN	46	-37	3	0.497	0.006	0.473	0.010
STS	VAN	47	-22	-9	0.379	0.042	0.465	0.011
STS	VAN	61	-39	2	0.382	0.041	0.418	0.024
**REACTION TIME**								
dlPFC	DMN	-42	16	48	0.443	0.016	0.520	0.004
FEF	DAN	-45	3	32	0.305	**0.107**	0.573	0.001
IPL	DMN	-47	-58	31	0.532	0.003	0.334	**0.076**
SMA	SM	5	-17	52	-0.536	0.003	-0.386	0.038
FG	VN	-34	-44	-22	-0.379	0.042	-0.459	0.012
IPS	DAN	-43	-45	43	0.238	**0.213**	0.624	0.0003
STS	VAN	58	-45	9	-0.297	**0.118**	-0.594	0.0007
SMA	FPN	-6	29	44	0.375	0.045	0.441	0.017
SMG	CON	58	-40	35	0.311	**0.101**	0.492	0.007

Abbreviations: BN: between-network, dlPFC: dorsolateral prefrontal cortex, FEF: frontal eye field, FG: fusiform gyrus, IPL: inferior parietal lobule, IPS: intraparietal sulcus, PHG: parahippocampal gyrus, SMA: supplementary motor area, SMG: supramarginal gyrus, STS: superior temporal sulcus, WN: within-network
Networks: CON: cingulo-opercular network, DAN: dorsal attention network, DMN: default mode network, FPN: frontoparietal network, SM: somatomotor network, UA: unassigned, VAN: ventral attention network
*Non-significant correlations are boldfaced.

SUPPLEMENTARY REFERENCES

1. Kay SR, Fiszbein A, Opler LA. The positive and negative syndrome scale (PANSS) for schizophrenia. *Schizophr Bull*. 1987;13(2):261-76. doi:10.1093/schbul/13.2.261

2. Andreasen NC, Pressler M, Nopoulos P, Miller D, Ho BC. Antipsychotic dose equivalents and dose-years: a standardized method for comparing exposure to different drugs. *Biol Psychiatry*. Feb 1 2010;67(3):255-62. doi:10.1016/j.biopsych.2009.08.040

3. Kucyi A, Esterman M, Capella J, et al. Prediction of stimulus-independent and task-unrelated thought from functional brain networks. *Nat Commun*. Mar 19 2021;12(1):1793. doi:10.1038/s41467-021-22027-0
